# Repeated Sprint Ability in Young Basketball Players (Part 2): The Chronic Effects of Multidirection and of One Change of Direction Are Comparable in Terms of Physiological and Performance Responses

**DOI:** 10.3389/fphys.2016.00262

**Published:** 2016-06-27

**Authors:** Giuseppe Attene, Pantelis T. Nikolaidis, Nicola L. Bragazzi, Antonio Dello Iacono, Fabio Pizzolato, Alessandro M. Zagatto, Juliano Dal Pupo, Marcello Oggianu, Gian M. Migliaccio, Elena Mannucci Pacini, Johnny Padulo

**Affiliations:** ^1^Italian Olympic CommitteeCagliari, Italy; ^2^Department of Physical and Cultural Education, Hellenic Army AcademyAthens, Greece; ^3^Department of Health Sciences, University of GenoaGenoa, Italy; ^4^Department of Life Sciences, Orde Wingate Institute for Physical Education and SportsNetanya, Israel; ^5^Department of Neurological and Movement Sciences, School of Exercise and Sport Science, University of VeronaVerona, Italy; ^6^Faculty of Sciences, Universidade Estadual PaulistaBauru, Brazil; ^7^Biomechanics Laboratory, Federal University of Santa CatarinaFlorianopolis, Brazil; ^8^Sport Science LabLondon, UK; ^9^University eCampusNovedrate, Italy; ^10^Faculty of Kinesiology, University of SplitSplit, Croatia

**Keywords:** exercise physiology, field testing, jump performance, rating of perceived exertion, shuttle running, training and testing

## Abstract

The aim of this study was to examine the effects of a 5-week training program, consisting of repeated 30-m sprints, on two repeated sprint ability (RSA) test formats: one with one change of direction (RSA) and the other with multiple changes of direction (RSM). Thirty-six young male and female basketball players (age 16.1 ± 0.9 years), divided into two experimental groups, were tested for RSA, RSM, squat jump, counter-movement jump, and the Yo-Yo Intermittent Recovery-Level-1 (Yo-Yo IR1) test, before and after a 4-week training program and 1 week of tapering. One group performed 30-m sprints with one change of direction (RSA group, RSAG), whereas the other group performed multidirectional 30-m sprints (RSM group, RSMG). Both groups improved in all scores in the post-intervention measurements (*P* < 0.05), except for the fatigue index in the RSM test. However, when comparing the two groups, similar effects were found for almost all parameters of the tests applied, except for RPE in the RSA test, which had a greater decrease in the RSAG (from 8.7 to 5.9) than in the RSMG (from 8.5 to 6.6, *P* = 0.021). We can conclude that repeated 30-m sprints, either with one change of direction or multidirectional, induce similar physiological and performance responses in young basketball players, but have a different psycho-physiological impact.

## Introduction

Repeated sprint ability (RSA) has been identified as a key component of physical fitness in team sports, especially in basketball (Bishop et al., 2001; Spencer et al., 2005; Padulo et al., 2015b, 2016). RSA has been defined as the ability to perform repeated sprints with minimal recovery (Stojanovic et al., 2012), or, in other words, the ability to produce the best possible average sprint performance over a series of sprints separated by short recovery periods (Bishop et al., 2001, 2011). Various RSA tests, differing in sprint distance, number of sprint repetitions, and duration of rests between repetitions and between different changes of direction (CODs), have been designed and extensively used to assess and monitor performance (Meckel et al., 2009; Stojanovic et al., 2012; Attene et al., 2015b; Padulo et al., 2015a). Recently, a novel sport-specific test that includes sprints performed forwards and backwards, to the left and backwards, and to the right and backwards (RSA—multidirectional; RSM) has been developed (Padulo et al., 2015b, 2016). Although these studies have improved the understanding of the acute physiological responses to the variety of RSA tests, there is a surprisingly limited body of research investigating their chronic effects.

The effectiveness of a long-term (>4 weeks) RSA training program has been reviewed by Bishop et al. (2011), and, since then, researchers have been collecting scientific evidences and investigating how to provide coaches with an objective approach for strategically selecting exercises and choosing the tactics that best generate chronic and optimal physiological conditions. However, few studies are available specifically concerning basketball.

The aim of this study was to examine the effects of a 4-week training program, consisting of repeated 30-m sprints, and 1 week of tapering, on two RSA test formats: one with one COD (RSA) and the other with multiple CODs (RSM). The main hypothesis of this research is that RSA, but not RSM, will result in performance improvements in the RSA group (RSAG), while RSM will lead to improvements in the RSM group (RSMG).

## Methods

### Participants

Thirty-six young (age 16.1 ± 0.9 years) male (*n* = 14) and female (*n* = 22) basketball players (see Table 1), who trained ~5 h per week, were recruited from the San Paolo Basket Cagliari. All the participants competed in the Under-17 Italian National Basketball Championship 2012–2013. Inclusion criteria to participate in the study were: (i) participation in at least 90% of the training sessions, (ii) regular participation in the previous competitive seasons, (iii) having a valid sport medical certificate, and (iv) being healthy (no pain or injury reported) and not receiving any medication or consuming other drugs. The participants refrained from drinking alcohol or beverages containing caffeine for 24 h, and did not eat for 2–3 h, prior to the testing sessions, to reduce any interference with the experimental results. To minimize any influence of circadian variation (Ammar et al., 2015), each participant completed all trials at the same time on the days of testing (2–4 p.m.) and with the same environmental conditions (22.2 ± 0.5°C temperature and 68.3 ± 3.5% relative humidity). All tests were performed on a regular indoor basketball court, and the participants wore basketball shoes and comfortable sportswear. Written consent was obtained from the participants' parents/guardians after being thoroughly informed of the purpose and potential risks of the study. All experimental procedures were approved by the University Human Research Ethics Committee of the CONI—Italian Olympic Committee, Sardinia, Cagliari, Italy, according to the ethical principles laid out in the 2008 revision of the Declaration of Helsinki.

**Table 1:** **Descriptive statistics of the participant's sample**.

**Variables**	**Training**	**Male**					**Female**					**M vs. F**			**Total**							
		***N***	**Mean**	***SD***	**95% CI**		***N***	**Mean**	***SD***	**95% CI**		***F***	**Sig**.	***ES***	***N***	**Mean**	***SD***	**95% CI**		***F***	**Sig**.	***ES***
Age [years]	RSAG	7	16.3	0.5	16.0	16.6	11	15.7	1.1	15.0	16.4	1.486	0.232	0.424	18	15.9	0.9	15.3	16.6	0.325	0.572	0.201
	RSMG	7	16.3	0.5	16.0	16.6	11	16.1	1.1	15.4	16.8				18	16.2	0.9	15.6	16.7			
	Total	14	16.3	0.5	16.0	16.6	22	15.9	1.1	15.2	16.6				36	16.1	0.9	15.5	16.6			
Stature [m]	RSAG	7	1.78	0.06	1.74	1.82	11	1.65	0.06	1.61	1.69	43.963	*P* < 0.0001	2.307	18	1.70	0.08	1.65	1.76	0.000	0.986	0.006
	RSMG	7	1.79	0.06	1.75	1.83	11	1.64	0.07	1.60	1.69				18	1.70	0.10	1.63	1.76			
	Total	14	1.78	0.06	1.75	1.82	22	1.65	0.06	1.61	1.69				36	1.70	0.09	1.64	1.76			
Weight[kg]	RSAG	7	64.7	6.2	60.7	68.7	11	57.0	6.4	52.8	61.2	17.398	*P* < 0.0001	1.451	18	60.0	7.2	55.3	64.7	0.001	0.977	0.010
	RSMG	7	66.4	6.0	62.5	70.3	11	55.4	7.4	50.6	60.2				18	59.5	8.7	53.8	65.1			
	Total	14	65.6	5.9	61.7	69.4	22	56.2	6.8	51.7	60.6				36	59.7	7.9	54.6	64.9			
BMI	RSAG	7	20.5	2.0	19.2	21.8	11	20.9	1.6	19.8	21.9	0.019	0.891	0.048	18	20.7	1.7	19.6	21.8	0.001	0.978	0.010
	RSMG	7	20.8	1.5	19.8	21.7	11	20.6	2.4	19.0	22.1				18	20.6	2.1	19.3	22.0			
	Total	14	20.6	1.7	19.5	21.7	22	20.7	2.0	19.4	22.0				36	20.7	1.9	19.4	21.9			

The table reports the age, stature, weight, and Body Mass Index (BMI) of each training group (RSAG, participants trained with RSA; RSMG, participants trained with RSM) and for the whole group (Total). Moreover, the F-value and the significant value (Sig.) of gender (M vs. F) and the between groups (Total) statistics are reported. For each variable the number of participants (N), the mean, the standard deviation, the 95% of confidence interval (95% CI), and the Cohen's d effect size (ES) are reported. The significant value (P < 0.05) is reported with^*^.

### Experimental setup

Two parallel groups were recruited and longitudinally tested. The participants were assigned to one of the two experimental groups—the RSAG or the RSMG, with randomized balanced blocks “one-to-one” (Schulz and Grimes, 2002). The overall study started after the first match of the second half of the competitive season (from February to April), and lasted 7 weeks. This period consisted of 1 week of tests (pre), 4 weeks of specific training (twice per week), 1 week of tapering (i.e., low intensity exercise based on technical skill, core stability exercise (Dello Iacono et al., 2016), and dynamic stretching (Chaouachi et al., 2015), and an additional week of tests (post). The training consisted of three sets of six maximal sprints (with 4 min of recovery between sets) in the first 2 weeks and eight maximal sprints in the second 2 weeks (with 20 s of recovery between sprints), while the distance for each sprint (30 m) was the same for RSA and RSM. The participants performed only basic basketball training; no strength and/or plyometric training was administered during the experimental period. In the two testing weeks the participants performed four randomized testing sessions: (i) RSA test; (ii) multiple CODs test; (iii) jump performances [squat jump (SJ) and countermovement jump (CMJ)]; and (iv) Yo-Yo Intermittent Recovery Level 1 (Yo-Yo IR1). The tests were separated by at least 2 days, and 1 week before the pre-test the RSA and multiple CODs tests were administered with the aim of assessing the reliability of the measures (Padulo et al., 2016).

Before each test, the participants completed a 10-min warm-up of low-intensity running (~8 km·h^−1^) and 5 min of standardized dynamic stretching (Chaouachi et al., 2015). For both tests (RSA and multiple CODs) the time for each single shuttle sprint was recorded using two photocell gates (Brower Timing System, Salt Lake City, UT, USA; accuracy of 0.01 s) placed at a height of 50 cm on the starting and finishing line, as standard procedure (Padulo et al., 2014). The participants were first familiarized with the test (i.e., they performed three sub-maximal 30-m shuttle sprints with 1 min of recovery in-between). After 5 min of recovery, a preliminary maximal single shuttle sprint was assessed as the criterion score for the subsequent tests, and the test started 5 min later.

### Repeated sprint ability (RSA) test

The RSA test (Padulo et al., 2015b) consisted of ten 30-m shuttle-sprints (15 + 15 m), each with a single COD of 180°, intercepted by 30 s of passive recovery, and with an exercise-to-rest ratio of 1:5 (Ruscello et al., 2013). The participants sprinted linearly from the start line for 15 m, touched a line on the floor with one foot, and then, following a 180° COD, returned to the start line as fast as possible.

### Repeated sprint multidirection (RSM) test

The multiple CODs test consisted of 10 repetitions of six shuttle sprints of 5 m (total 30 m) in three directions, with five CODs, spaced out by 30 s of passive recovery consisting of walking back to the starting line and waiting for the next shuttle sprint (Padulo et al., 2016). To balance the legs' effort, the participants were asked to alternate the leg used for each COD in random order for the first sprint (Pau et al., 2014).

For both tests (RSA and RSM) the fatigue index (FI) was calculated using the Fitzsimons formula [100 × (TT / (BT × 10)) − 100], where TT corresponds to total time and BT to the best time (Fitzsimons et al., 1993). The multiple CODs have a strong criterion validity measured against a benchmark test (RSA), and good reliability and a high correlation with the SJ, the CMJ, and the Yo-Yo IR1 test (Padulo et al., 2016).

### Jump performance tests

The SJ and CMJ were explained and demonstrated to each participant, and familiarization trials were undertaken until the correct technique (Gheller et al., 2015) was acquired (as judged by the experimenter). These tests were performed in randomized order (Latin square design) according to the protocol described by Bosco and Rusko (1983). All players performed three repetitions of both the SJ test and the CMJ test, with a passive recovery of 1 min in-between, and at least 3 min between the two tests. The accelerometer Myotest™ (Myotest SA, Sion, Switzerland) was used to evaluate the jump height (cm) (Choukou et al., 2014).

For the SJ, participants started from an upright standing position with their hands on their hips; they were then instructed to flex their knees and hold a predetermined knee position (knee angle of ~90°). On the count of three, participants were asked to jump as high as possible without performing any counter-movement before the execution of the jump (Gheller et al., 2015). For the CMJ, participants started from an upright standing position with their hands on their hips (Attene et al., 2015a). They were instructed to quickly flex their knees (knee angle of ~90°) and then immediately jump as high as possible (Asmussen and Bonde-Petersen, 1974). During the jump, the trunk was kept as vertical as possible to limit its influence on performance. The participants were asked to repeat the test if the experimenter judged the jump to be performed incorrectly.

### Yo-Yo intermittent recovery test (Yo-Yo IR1)

All participants were already familiar with the Yo-Yo IR1 (Attene et al., 2014) testing procedures, since this test was part of their usual fitness assessment program. The test consisted of 20-m shuttle runs performed at increasing velocities until exhaustion, with 10 s of active recovery between runs. The test was considered to be over when the participant failed twice to reach the finishing line in time (objective evaluation), or when the athlete felt unable to complete another shuttle at the dictated speed (subjective evaluation). The total distance covered during the test (including the last incomplete shuttle) was considered as the test score.

### Rating of perceived exertion and blood lactate assessment

The participants indicated their rating of perceived exertion (RPE, CR10-scale modified by Foster et al., 2001) immediately at the end of the following tests: the RSA, RSM, and Yo-Yo IR1, and after each training session. The scores were reported after the participants had reached their same heart-rate as in the starting condition, firstly on a paper grid and then reported in a data file. The blood lactate concentration ([La^−^] mmol·L^−1^) was determined from capillary blood samples obtained from the ear lobe at the 3rd minute after completion of the RSA, RSM, and Yo-Yo IR1 tests, as reported in Hirvonen et al. (1987). The blood sample was analyzed immediately, using a lactate analyzer (Arkray Lactate Pro LT-1710 Kyoto, Japan) that had been previously calibrated.

### Training program

During the regular basketball season the participants trained three times a week for almost 90 min at each session. Two training sessions were dedicated to the experimental training, which was performed at the same time by the two groups (RSAG and RSMG), and never performed on two consecutive days. The session started with a 15-min warm-up (5 min of low-intensity running, 5 min of dynamic stretching, and 5 min of skipping), followed by the experimental training. The remaining training time was dedicated to specific basketball training, which was the same for both groups. The participants were asked to sprint at their maximal speed and effort for each shuttle run. The training volume was similar for both groups, modified only by the number of CODs required by the study protocol (i.e., RSA or RSM). At the end of the experimental training sessions the Borg category ratio RPE scale was assessed, in order to calculate the subjective working load (SWL).

### Statistical analysis

Data are reported as mean ± standard deviation (SD), 95% of confidence interval. Prior to each statistical analysis the homogeneity of variance was verified with Bartlett's test, and the normality of distribution of each variable was tested with the Shapiro–Wilk test.

A multi-factor repeated measure analysis of variance (ANOVA) was performed to investigate the impact of time, training intervention (RSAG and RSMG), and gender on each variable. Finally, the SWL was computed following the equation developed by Foster et al. (2001): training duration in minutes multiplied by the mean RPE training intensity and normalized with respect to the first day of training. Finally, a two-way repeated measures ANOVA was conducted to discover group differences within the training period. The within factors were weeks of training (four levels, 4 weeks), and repetitions (two per week). The between factor was the groups (RSAG, RSMG). Moreover, for each statistical analysis of variance the Cohen's *d* effect size (ES) was calculated, defined as the difference between two means divided by SD for the data; the magnitude of the effect was interpreted based on the Cohen's rule of thumb, namely, ranges 0–0.1 no effect, 0.2–0.4 small effect, 0.5–0.7 intermediate effect, and >0.8 large effect (Cohen, 1988).

Statistical analysis was performed using SPSS 22.0 (SPSS Inc., Chicago, IL, USA). For all the performed analyses, a *P*-value < 0.05 was considered significant.

## Results

The results of the statistics: *F*-value, *P*-value and the Cohen's *d* ES are reported in the tables. Descriptive statistics of the participant's samples are presented in Table 1. The weight and stature were higher for males compared to females, with a large effect size, while there were no differences in effects between the training groups' age and BMI (Table 1). Four weeks of training followed by 1 week of tapering allowed for an improvement in performance for all the measured variables (time effect; see Tables 2, 3), except for the FI at the RSA test. In particular, at the RSA test (Table 2), ES was small for BT, intermediate for WT and TT, large for lactate and RPE. Only lactate concentration showed a time × gender effect (small effect), whereas lactate concentration and RPE exhibited a small effect when taking into account the interaction time × gender × group. In more details, lactate concentration was lower among females than among males, even though this statistically significant difference tended to attenuate over time. RPE was lower among females, of the RSAG group with respect to other groups, with this difference becoming statistically significant at the end of the training period. At the RSM test (Table 3), all studied variables exhibited an intermediate effect considering time, but the FI which was characterized by a small effect, while lactate concentration showed a small effect considering time × gender interaction.

**Table 2:** **Statistics of the RSA test**.

**Variable**	**Training**	**Pre**				**Post**				**Time effect**			**Time** × **Gender effect**			**Time** × **Group effect**			**Time** × **Gender** × **Group effect**		
		**Mean**	***SD***	**95% CI**		**Mean**	***SD***	**95% CI**		***F***	**Sig**.	***ES***	***F***	**Sig**.	***ES***	***F***	**Sig**.	***ES***	***F***	**Sig**.	***ES***
BT [s]	RSAG	6.451	0.496	6.127	6.774	6.263	0.545	5.907	6.620												
	RSMG	6.368	0.350	6.140	6.597	6.227	0.473	5.918	6.536												
	Total	6.411	0.427	6.132	6.689	6.246	0.504	5.916	6.575	11.85	0.002	0.26	0.53	0.47	0.02	0.16	0.69	0.01	0.02	0.90	0.00
WT [s]	RSAG	7.179	0.518	6.841	7.517	6.698	0.564	6.329	7.066												
	RSMG	7.020	0.390	6.765	7.275	6.674	0.435	6.389	6.958												
	Total	7.102	0.461	6.801	7.403	6.686	0.498	6.361	7.011	64.56	<0.001	0.66	4.33	0.045	0.12	2.26	0.14	0.06	0.00	0.96	0.00
TT [s]	RSAG	67.68	5.125	64.33	71.03	64.74	5.543	61.12	68.36												
	RSMG	66.84	3.660	64.45	69.23	64.48	4.417	61.60	67.37												
	Total	67.27	4.429	64.38	70.16	64.62	4.956	61.38	67.85	48.00	<0.001	0.59	2.98	0.094	0.08	0.28	0.60	0.01	0.84	0.37	0.03
Lactate [mmol·L^−1^]	RSAG	10.2	1.2	9.4	10.9	8.2	1.3	7.4	9.0												
	RSMG	8.9	1.8	7.7	10.1	7.9	1.5	6.9	8.9												
	Total	9.5	1.6	8.5	10.6	8.0	1.4	7.2	8.9	91.16	<0.001	0.83	5.59	0.029	0.23	3.55	0.075	0.16	5.12	0.035	0.21
FI	RSAG	−11.4	4.8	−14.5	−8.3	−7.0	2.4	−8.6	−5.4												
	RSMG	−10.3	3.7	−12.7	−7.9	−7.3	3.8	−9.8	−4.8												
	Total	−10.9	4.3	−13.7	−8.1	−7.1	3.1	−9.2	−5.1	3.56	0.068	0.10	1.01	0.32	0.03	2.05	0.16	0.06	0.00	0.96	0.00
RPE	RSAG	8.7	0.5	8.4	9.0	5.9	0.7	5.5	6.4												
	RSMG	8.5	0.5	8.1	8.8	6.6	0.5	6.3	6.9												
	Total	8.6	0.5	8.3	8.9	6.3	0.7	5.815	6.718	373.08	<0.001	0.92	4.71	0.037	0.13	5.18	0.029	0.14	8.47	0.006	0.20

*For all the variables of the RSA test, mean and standard deviation (SD) and 95% of confidence interval (95% CI) are reported, divided by groups trained with RSA (RSAG) and RSM (RSMG), and also the general improvement of the two groups together (Total). For each variable (BT, Best Time; WT, Worst Time; TT, Total Time; Lactate, Blood Lactate; FI, Fatigue Index; RPE, Rating of Perceived Exertion) the initial result (Pre), and the results after 4 weeks of training (Post) are reported. For the analysis the F-value and the P-value, and the Cohen's d effect size (ES) are reported*.

**Table 3:** **Statistics of the RSM test**.

**Variable**	**Training**	**Pre**				**Post**				**Time effect**			**Time** × **Gender effect**			**Time** × **Group effect**			**Time** × **gender** × **group effect**		
		**Mean**	***SD***	**95% CI**		**Mean**	***SD***	**95% CI**		***F***	**Sig**.	***ES***	***F***	**Sig**.	***ES***	***F***	**Sig**.	***ES***	***F***	**Sig**.	***ES***
BT [s]	RSAG	10.199	0.748	9.710	10.688	9.691	0.715	9.224	10.158												
	RSMG	10.296	0.656	9.867	10.725	9.781	0.579	9.403	10.159												
	Total	10.246	0.697	9.791	10.701	9.735	0.644	9.314	10.156	70.06	<0.001	0.69	0.03	0.86	0.00	0.02	0.89	0.00	2.95	0.10	0.08
WT [s]	RSAG	11.255	0.825	10.716	11.794	10.498	0.785	9.986	11.011												
	RSMG	11.308	0.667	10.872	11.743	10.561	0.632	10.148	10.974												
	Total	11.281	0.742	10.796	11.765	10.529	0.705	10.068	10.989	90.69	<0.001	0.74	2.47	0.13	0.07	0.06	0.81	0.00	0.90	0.35	0.03
TT [s]	RSAG	106.84	7.408	102.00	111.68	100.85	7.338	96.06	105.64												
	RSMG	107.69	6.628	103.36	112.02	101.52	5.650	97.83	105.21												
	Total	107.25	6.950	102.71	111.79	101.18	6.485	96.94	105.41	98.29	<0.001	0.75	0.00	0.97	0.00	0.01	0.91	0.00	2.84	0.10	0.08
Lactate [mmol·L^−1^]	RSAG	9.3	1.6	8.3	10.3	8.0	1.4	7.1	8.9												
	RSMG	8.3	1.6	7.3	9.3	7.6	1.4	6.7	8.5												
	Total	8.8	1.6	7.7	9.8	7.8	1.4	6.9	8.7	63.26	<0.001	0.76	9.35	0.006	0.32	2.66	0.12	0.12	3.39	0.08	0.15
FI	RSAG	−10.4	4.0	−13.0	−7.8	−8.3	2.1	−9.7	−6.9												
	RSMG	−9.9	4.7	−13.0	−6.9	−8.0	3.6	−10.4	−5.7												
	Total	−10.2	4.3	−13.0	−7.4	−8.2	2.9	−10.1	−6.3	11.79	0.002	0.27	4.92	0.034	0.13	0.03	0.87	0.00	0.26	0.62	0.01
RPE	RSAG	8.0	1.1	7.3	8.7	6.9	1.1	6.2	7.6												
	RSMG	7.9	1.1	7.2	8.6	6.9	0.9	6.3	7.5												
	Total	7.9	1.0	7.2	8.6	6.9	1.0	6.2	7.5	36.12	<0.001	0.57	1.09	0.31	0.04	0.58	0.45	0.02	0.23	0.64	0.01

*For all the variables of the RSM test the mean and standard deviation (SD) and 95% of confidence interval (95% CI), divided by groups trained with RSA (RSAG) and RSM (RSMG), and also the general improvement of the two groups together (Total), are reported. For each variable (BT, Best Time; WT, Worst Time; TT, Total Time; Lactate, Blood Lactate; FI, Fatigue Index; RPE, Rating of Perceived Exertion) the initial result (Pre), and the results after 4 weeks of training (Post) are reported. For the analysis the F-value and the P-value and the Cohen's d effect size (ES) are reported (repeated measures ANOVA)*.

In addition, both training types were able to improve the SJ and CMJ with a small effect, as well as the Yo-Yo IR1 total distance, with a large effect concerning time and with a small effect considering time × gender interaction, while the Yo-Yo IR1 RPE was not influenced by the training (Table 4). The training comparison (see Tables 2–4) showed that neither type of training produced an effect in the RSA and RSM tests. Only RPE was significantly smaller in the RSMG (from 8.5 to 6.6) (see Table 3) compared to the RSAG (from 8.7 to 5.9) (see Table 2), although having a very small ES.

**Table 4:** **Statistics of jump tests and the Yo-Yo test**.

**Variable**		**Pre**				**Post**				**Time effect**			**Time** × **Gender effect**			**Time** × **Group effect**			**Time** × **Gender** × **Group effect**		
	**Traning**	**Mean**	***SD***	**95% CI**		**Mean**	***SD***	**95% CI**		***F***	**Sig**.	***ES***	***F***	**Sig**.	***ES***	***F***	**Sig**.	***ES***	***F***	**Sig**.	***ES***
SJ [cm]	RSAG	28.1	7.8	23.0	33.1	29.5	7.5	24.6	34.4												
	RSMG	27.9	8.5	22.4	33.5	29.9	8.3	24.4	35.3												
	Total	28.0	8.0	22.8	33.2	29.7	7.8	24.6	34.8	30.74	<0.001	0.48	0.00	0.97	0.00	0.72	0.40	0.02	0.79	0.38	0.02
CMJ [cm]	RSAG	29.6	8.5	24.1	35.1	31.3	7.8	26.2	36.3												
	RSMG	29.2	8.8	23.5	35.0	31.0	8.4	25.5	36.5												
	Total	29.4	8.5	23.9	35.0	31.1	8.0	25.9	36.3	23.31	<0.001	0.41	0.98	0.33	0.03	0.27	0.61	0.01	0.48	0.49	0.01
Yo-Yo distance [m]	RSAG	1105	314	900	1310	1435	376	1190	1681												
	RSMG	1092	238	936	1247	1441	271	1264	1618												
	Total	1098	274	919	1277	1438	323	1227	1649	138.39	<0.001	0.81	8.54	0.006	0.21	0.04	0.85	0.00	2.33	0.14	0.07
Yo-Yo RPE	RSAG	9.4	0.5	9.1	9.7	9.4	0.5	9.1	9.7												
	RSMG	9.3	0.6	8.9	9.7	9.2	0.6	8.9	9.6												
	Total	9.4	0.5	9.0	9.7	9.3	0.5	9.0	9.7	0.00	0.96	0.00	0.47	0.50	0.01	0.47	0.50	0.01	0.00	0.96	0.00

*Results of the Squat Jump test (SJ), Countermovement Jump (CMJ) and Yo-Yo Intermittent Recovery test level 1 distance (Yo-Yo distance) and Rating of Perceived Exertion of the Yo-Yo test (Yo-Yo RPE) divided by groups trained with RSA (RSAG) and RMS (RSMG), and also the general improvement of the two groups together (Total), are reported. For each variable mean and standard deviation (SD) and 95% of confidence interval (95% CI) of the initial result (Pre), and the results after 4 weeks of training (Post) are reported. For the analysis, the F-value, the P-value and the Cohen's d effect size (ES) are reported (repeated measures ANOVA)*.

## Discussion

The main finding of the present study was that training consisting of repeated 30 m sprints, performed either with one COD or multidirectional, resulted in similar changes in the RSA performance as well as in the aerobic (Yo-Yo IR1 test) and neuromuscular (SJ, CMJ) parameters analyzed. Thus, the research hypothesis was not confirmed. The only different effect between the two groups was a smaller decrease of RPE in the RSA test for the RSMG than for the RSAG.

**Figure 1:**
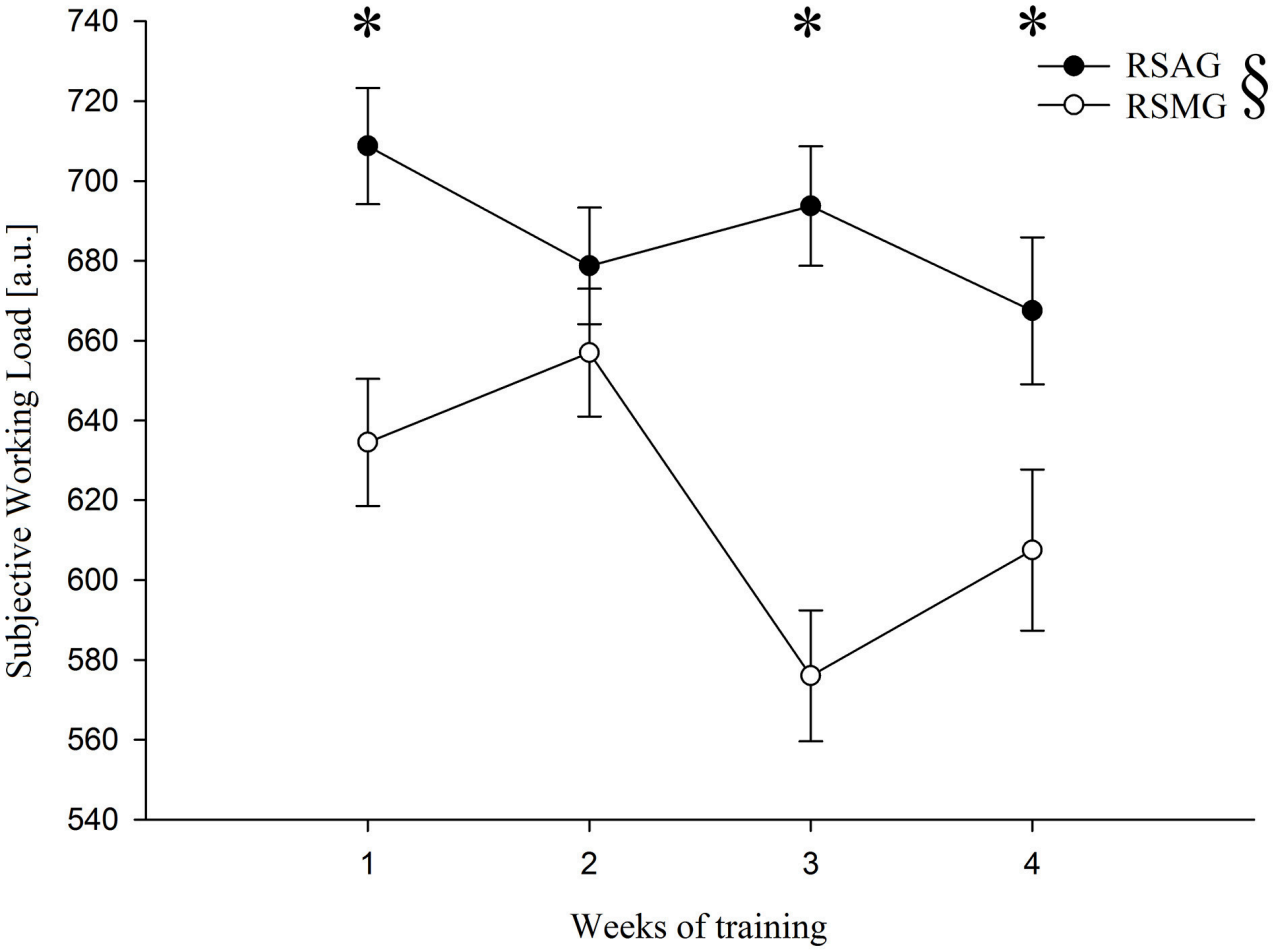
**Subjective working load (in arbitrary units, a.u.) for the two groups § (RSAG—that is to say participants trained with RSA, and RSMG—that is to say participants trained with RSM) over weeks of training**. ^*^Statistically significant with *P*-value < 0.05.

The training program of the two groups (Figure 1) differed with regard to two parameters. Firstly, the multidirectional mode and the higher number of CODs in the RSMG imposed higher muscular demands compared to the RSAG. Secondly, the work-to-rest ratio differed for the RSA and RSM tests, as well as for the two training programs. This ratio was 1/5 for the RSA and 1/4 for the multiple CODs test, and 1/4 for the RSAG and 1/3 for RSMG training. This variation in the work-to-rest ratio was attributed to the longer duration of a multidirectional sprint than the duration of a sprint with a COD (10.73 vs. 6.73 s, respectively), and to the shorter duration of rest between sprints in training than in testing (20 vs. 30 s, respectively). In this context, a study by Ben Abdelkrim et al. (2007) highlighted the dominance of activities of 2-s duration normally seen in competition, with about 1050 intermittent an repeated actions during a 40-min basketball game. Moreover, due to the high-intensity intermittent nature of basketball, players may be required to reproduce maximal or near-maximal short duration efforts with only brief recovery periods (<20 s consisting of complete rest or low- to moderate-intensity activity). Thus, it was concluded that basketball players, in addition to having good RSA, also have to perform many CODs—approximately every 3 s during the play, with very short recovery periods. Therefore, relying on the physiological and time-motion analysis (Ben Abdelkrim et al., 2007) responses recorded during official matches (average actions ~2.5 s with short recovery <20 s), and referring to the optimal training regimes required to improve physical abilities of basketball players (Attene et al., 2014, 2015a), both prescribed protocols represented an efficient training stimulus. However, regimens including multidirectional sprint should be preferred, due to their higher similarities to the performance model of the discipline of interest.

In the current study, there was no reference data to compare the participants' performance with the new multiple CODs test; however, in other studies a similar RSA test protocol [i.e., 10 × (15 + 15-m)] was used for male basketball players—elite adults (Stojanovic et al., 2012) and juniors ~16.5 years (Caprino et al., 2012). In the total sample, the TT of the RSA test improved by −4% (from 67.27 to 64.62 s). The initial score was lower than that of the adults (58-s, (Stojanovic et al., 2012)) or juniors (62 s). The FI also improved, by +30.1% (from −10.9 to −7.1%), which was less than in the abovementioned studies (3.4–3.5%, Stojanovic et al., 2012). Lactate concentration decreased by −18.3% (from 9.5 to 8.0 mmol·L^−1^), which was higher than that of juniors (12.4 < 14.2 mmol·L^−1^ Caprino et al., 2012). A high blood lactate concentration would imply high activation of anaerobic glycolysis. A previous study (Balsom et al., 1992) showed that lactate concentration was higher for sprint repetitions of a shorter distance.

The overall improvement of time variables in the RSA and multiple CODs tests for both training groups in our study was accompanied by a respective increase in jumping performance. SJ and CMJ increased similarly after repeated sprint training with one or multidirectional COD. In the present study, the CMJ increased in both groups by ~7%, which was similar to or slightly lower than that reported in previous experimental studies. For instance, a ~10% increase of jumping performance after a 34-week period was observed by Santos et al. (2014). In another study, investigating the effects of a 10-week resistance training for the upper and lower body on explosive strength indicators in male basketball players (age ~14.5 years), the experimental group improved by 12.5% in the SJ and 10.2% in the CMJ, whereas the control group decreased its respective values (Santos and Janeira, 2012). Moreover, in an investigation including a supervised 6-week program in female basketball players (age 15.5 years), 3 days weekly, a significant improvement was found in the vertical jump (Abalakov) test +9% (Noyes et al., 2012). Finally, adult male basketball players participated in a 10-week plyometric training program in which the unloaded plyometric group improved the SJ and CMJ by 6 and 7%, respectively, and the loaded plyometric group improved by 10 and 12%, respectively, whereas no change was observed in a control group (Khlifa et al., 2010).

The relationship between sprint and jumping performance in basketball has been shown by both observational (Shalfawi et al., 2011) and experimental (Tsimahidis et al., 2010) studies. An observational study on adult male basketball players showed large to very large correlations of SJ and CMJ with 30 m sprint time (Alemdaroglu, 2012). Furthermore, an observational study on adult male basketball players showed moderate to large correlations of SJ and CMJ with 10-m and 20-m sprint times, and a very large correlation of SJ and CMJ with a 40-m time (Shalfawi et al., 2011). In line with these observational studies, the relevant experimental research has shown an increase in both sprint and jumping performance after exercise interventions. In a study on the effect of a 6-week training program on vertical jump of young male basketball players (age 20 years), the group that trained with plyometrics twice weekly improved their vertical jump by ~24% and 4 × 9 m sprint time by ~7%, whereas there was no change in the control group (Asadi, 2013). In study reporting on a 10-week heavy resistance combined with a running training program in basketball players (age 18 years), the experimental group improved their 10 and 30 m sprint time, SJ, and CMJ, whereas no changes in the control group were shown (Tsimahidis et al., 2010).

In the current study, an overall improvement of ~33% was observed in the Yo-Yo test (from 1098 to 1438 m). Despite our hypothesis, we were not able to observe any differences in improvement in the Yo-Yo test between RSAG and RSMG. A recent study (da Silva et al., 2015) also investigated the effects of two different aerobic training models (straight line and shuttle-run), in this case on the aerobic fitness of soccer players. The authors hypothesized that there would be great improvements in the group with the directional changes, but the results showed that the two modes of training improved the indices of aerobic power (velocity at VO_2max_) and capacity (anaerobic threshold) similarly in the players. Thus, despite the high intensity induced by the CODs requiring additional muscular actions for decelerations/accelerations, it seems that the COD training did not induce greater aerobic adaptation. In an earlier study (Balsom et al., 1992), it was shown that post-test VO_2_ was higher for sprint repetitions of longer distances, indicating a higher oxygen debt and, consequently, a higher contribution of the anaerobic energy transfer system.

## Limitations

Our study has some limitations. The basketball players' training volume in addition to the two specific training session per week could have been controlled so that RSAG and RSMG would receive similar training volumes. Furthermore, another limitation is the limited sample size. As such, caution should be taken when generalizing the findings of this study.

## Conclusions

This study investigated the effects of a 5-week training program consisting of repeated 30-m sprints, on two RSA test formats—one with one COD and the other with multiple CODs—in 36 young basketball players allocated to one of the two experimental groups (RSAG and RSMG). Both groups improved in all scores in the post-intervention measurements, even though no difference could be detected for any of the investigated parameters except for the fatigue index in the RSM test, which decreased more in the RSAG, even though with a little ES. We can conclude that repeated 30-m sprints induce similar physiological and performance responses in young basketball players, but have a different psycho-physiological impact in terms of fatigue. On the other hand, the training with multiple CODs reproduces more closely the kinds of movements typical of basketball and should be preferred from an ecological point of view, also considering that this type of training requiring a less training workload produces results comparable to the other type of training.

## Author contributions

JP conceived the experiment. GA, PN, AD, AZ, JD, MO, GM, EM collected the data. NB and FP analyzed the data. All authors wrote and approved the manuscript.

### Conflict of interest statement

The authors declare that the research was conducted in the absence of any commercial or financial relationships that could be construed as a potential conflict of interest.
